# Withaferin A protects against spinal cord injury by inhibiting apoptosis and inflammation in mice

**DOI:** 10.1080/13880209.2017.1288262

**Published:** 2017-02-23

**Authors:** Xianlei Yan, Guangxiang Huang, Quan Liu, Jiemin Zheng, Hongmou Chen, Qidan Huang, Jiakang Chen, Heqing Huang

**Affiliations:** a Department of Neurosurgery, the Fourth Affiliated Hospital of Guangxi Medical University, Liuzhou, China;; b Department of Neurosurgery, The First Affiliated Hospital of Guangxi Medical University, Nanning, China

**Keywords:** Central nervous system, natural product, pro-inflammatory cytokine, anti-inflammatory cytokine

## Abstract

**Context:** Withaferin A (WFA) exhibits diverse pharmaceutical applications on human diseases, including rheumatoid arthritis, cancers and microbial infection.

**Objective:** We evaluated the neuroprotective role of WFA using a mouse model of spinal cord injury (SCI).

**Materials and methods:** BALB/c mice were administrated 10 mg/kg of WFA. Gene expression was measured by real-time PCR, western blot and immunohistochemistry. Cell morphology and apoptosis were determined by H&E staining and TUNEL assay. Motor function was evaluated by the BBB functional scale for continuous 7 weeks.

**Results:** WFA significantly improved neurobehavioural function and alleviated histological alteration of spinal cord tissues in traumatized mice. Brain-derived neurotrophic factor (BDNF) and glial cell line-derived neurotrophic factor (GDNF) significantly increased in WFA-treated mice. Meanwhile, the expression of Nogo-A and RhoA remarkably decreased in the presence of WFA. Furthermore, the apoptotic cell death was attenuated in mice treated with WFA (31.48 ± 2.50% vs. 50.08 ± 2.08%) accompanied by decreased bax and increased bcl-2. In addition, WFA decreased the expression of pro-inflammatory mediators such as IL-1β (11.20 ± 1.96 ng/mL vs. 17.59 ± 1.42 ng/mL) and TNF-α (57.38 ± 3.57 pg/mL vs. 95.06 ± 9.13 pg/mL). The anti-inflammatory cytokines including TGF-β1 (14.32 ± 1.04 pg/mL vs. 9.37 ± 1.17 pg/mL) and IL-10 (116.80 ± 6.91 pg/mL vs. 72.33 ± 9.35 pg/mL) were elevated after WFA administration.

**Discussion and conclusion:** This study demonstrated that WFA has a neuroprotective role by inhibition of apoptosis and inflammation after SCI in mice.

## Introduction

Traumatic spinal cord injury (SCI) is one of the most common causes of long-term disability among young adults worldwide, which leads to neurological deficits and motor and sensory dysfunctions (Dietz & Fouad 2014; Filli & Schwab 2015). It is estimated that about 270,000 SCI cases were reported in the United States in 2012, most of them younger than 30 years old (Martin et al. 2015). Currently, there are no effective pharmacological treatments of SCI because of the functional and structural complexity of the spinal cord. Therefore, it is urgent to develop novel and effective therapeutic options for SCI.

Natural products serve as an excellent source for discovery and development of a substantial fraction of therapeutic drugs. The plant-derived natural compounds exhibit a wide range of applications on human diseases, including rheumatoid arthritis, cancers and microbial infection (Newman & Cragg 2012; Elshahawi et al. 2013). Withaferin A (WFA), a 28-carbon steroidal lactone, is a bioactive component of *Withania somnifera* (L.) Dunal (Solanaceae) and shows protective roles against infectious burns and dermatological diseases (Essawi & Srour 2000). Accumulating evidence suggests that WFA exhibits a diverse range of biological properties, such as anti-inflammatory, anti-angiogenic and anti-oncogenic effects (Yokota et al. 2006; Stan et al. 2008; Peng et al. 2010). On the molecular level, WFA modulates a variety of biological processes (e.g., cell growth, apoptosis, invasion and migration) by regulation of Akt/mTOR, Notch2/4 and NF-κB (Lee et al. 2012; Samadi et al. 2012; Grogan et al. 2014; Jackson et al. 2015). A recent study suggests that pre- or post-treatment of astrocytes with WFA can attenuate NF-κB activity by stimulation of the LPS/TLR4 pathway, suggesting that WFA may be an eligible candidate for the treatment of neuroinflammatory stress conditions (Martorana et al. 2015). However, the neuroprotective effects of WFA on SCI have never been investigated. Therefore, our present study aimed to explore the protective role of WFA using a mouse model of SCI.

## Materials and methods

### Animals

BALB/c mice weighing 18–22 g were maintained at the same centre and were given free access to food and water. This study was performed in accordance with the Guidance Suggestions for the Care and Use of Laboratory Animals made by the Ministry of Science and Technology of China and approved by the institutional animal care and research committee.

A total of 24 BALB/c mice were randomly divided into three groups (*n* = 8): control group, SCI group and WFA group. According to the previous report, SCI model was established according to Allen’s advanced method. Control animals were injected with an equal volume of sterile saline. Additional mice were treated intraperitoneally with 10 mg/kg of WFA 1 h before the contusion injury.

### Behavioural analysis

All the mice were evaluated by two experienced examiners via a double-blind method. The Basso, Beattie and Bresnahan (BBB) locomotor rating scale was used to determine the changes in motor function.

### Real-time PCR

Total RNAs were extracted by using TRIzol reagent (Invitrogen) and converted to cDNA using reverse transcription Kit (Promega, Madison, WI) according to the manufacturer’s protocol. Primers used or gene amplification were as follows.

BDNF forward:

5′-ACTATGGTTATTTCATACTTCGGTT-3′;

BDNF reverse: 5′-CCATTCACGCTCTCCAGA-3′.

GDNF forward:

5′-AAGGTCACCAGATAAACAAGGG-3′;

GDNF reverse: 5′-TCACAGGAGCCGCTGCAATATC-3′.

GAPDH forward: 5′-GGGGCTCTCTGCTCCTCCCTG-3′;

GAPDH reverse: 5′-CCAGGCGTCCGATACGGCCA-3′.

Real-time PCR was conducted using the SYBR Green PCR Master Mix (Toyobo, Japan) according to the instruction by the manufacture. Amplification protocols were followed: 95 °C for 10 min, 40 cycles of 95 °C/15 s, 55 °C/60 s and 72 °C/30 s. The transcript levels of interest genes were normalized to the GAPDH and were calculated with 2^−ΔΔ^*^C^*^t^ method.

### Histological examination

Mice were anesthetized and sacrificed at 24 h after injury, followed by treatment with 4% paraformaldehyde. Spinal cord tissues were immediately removed and post-fixed in 4% paraformaldehyde for 24 h. Paraffin-embedded sections with the thickness of 5 mm were stained with haematoxylin and eosin (H&E) for visualization under a light microscope (Leica Microsystems, Wetzlar, Germany) at 200 × magnification.

### TUNEL assay

Apoptotic cells in the spinal cord tissues were detected using the terminal deoxynucleotidyl transferase-mediated dUTP nick end labeling (TUNEL) according to the manufacturer’s instructions (Roche, Germany). The sections were deparaffinized, rehydrated and incubated with the terminal deoxynucleotidyl transferase enzyme at 37 °C for 2 h. Slides were stained with diaminobenzine, counterstained with haematoxylin and counted under a microscope (Olympus, Germany).

### Western blot

The protein was extracted from lung tissues and subjected to sodium dodecyl sulphate polyacrylamide gel electrophoresis (SDS-PAGE). After immunoblotting, membranes were transferred to nitrocellulose membranes (Amersham Pharmacia Biotech, Bucks, UK). Primary antibodies including anti-bax, bcl-2 and GAPDH (Santa Cruz, CA) were used for incubation with the membranes followed by the horseradish peroxidase-conjugated secondary antibodies. Protein expression was visualized using the ECL detection kit (Pierce Chromatography Cartridges, Rockford, IL). GAPDH was used as a loading control.

### Immunohistochemistry

The tissue sections were washed, blocked and incubated with the primary antibody at 4 °C for 12 h. After being washed three times in PBS, the slices were incubated with the secondary antibody at room temperature for 2 h. The sections were again washed with PBS, followed by incubation with 3,3′-diamino-benzidine chromogen solution. BDNF- and GDNF-positive cells were counted under a microscope (Leica, Germany).

### Enzyme-linked immunosorbent assay

The expression levels of cytokines including TGF-β1, IL-1β, IL-10 and TNF-α were determined using enzyme-linked immunosorbent assay (ELISA) according to the manufacturer’s instructions (R&D Systems, Minneapolis, MN). Absorbance was measured at 450 nm by microplate assay.

### Statistical analysis

For statistical analysis, all the data were presented as means ± SD of at least triplicate determinations and treated for statistics analysis by SPSS program. Comparison between groups was made using ANOVA. *p* < 0.05 was considered as statistically significant.

## Results

### WFA injection improved neurobehavioural function

**Figure 1.**
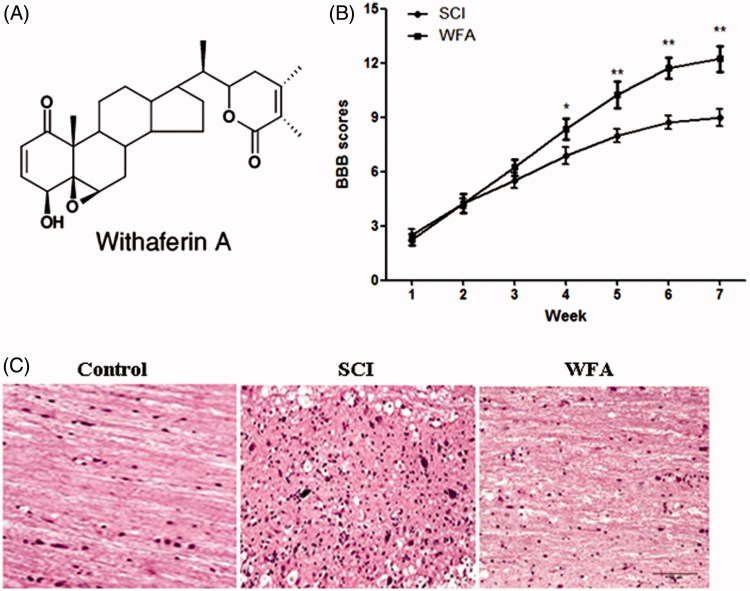
WFA treatment improved neurobehavioral function in mice. (A) Structure of WFA. Determination of BBB scores (B) and histological alterations (C) in SCI mice treated with saline or WFA (10 mg/kg). *p < 0.05, **p < 0.01, compared with SCI mice.

In order to evaluate the protective effects of WFA on neurobehavioural function, mice were treated with or without WFA prior to establishment of SCI model. And, the neurobehavioural tests were performed once a week for 7 weeks. Consequently, we found no obvious differences of BBB score between the SCI and WFA groups during the first 3 weeks. However, BBB scores were remarkably higher in the animals receiving WFA than those in SCI group from the fourth week (Figure 1(A,B)). No significant changes in BBB scores were observed in control group. The accelerated recovery of locomotion in the WFA-treatment group was maintained until week 7, suggesting that WFA exerted a protective role on neurobehavioural function in traumatized mice. In addition, a significant damage to the spinal cord from SCI mice was assessed by the presence of oedema and histological alteration, which was alleviated by WFA treatment (Figure 1(C)).

### WFA promoted the expression of BDNF and GDNF in spinal cord

**Figure 2.**
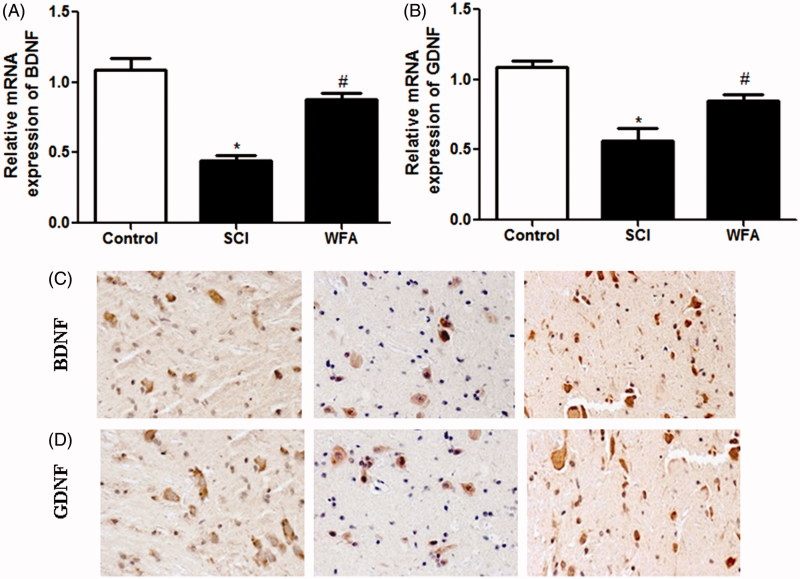
WFA promoted the expression of BDNF and GDNF in spinal cord. Real-time PCR and immunohistochemistry were performed to measure the mRNA- and protein expression of (A and C) BDNF and (B and D) GDNF in spinal cord tissues in SCI mice treated with saline or WFA (10 mg/kg). *p < 0.05, compared with control mice; #*p* < 0.05, compared with SCI mice.

To explore the effect of WFA injection on the expression of neurotrophic genes, real-time PCR analysis and immunohistochemistry were applied to measure the expression levels of GDNF and BDNF in spinal cord tissues. Compared to mice in the control group, traumatized mice showed reduced expression of BDNF. Nevertheless, animals injected by WFA showed significant increase in BDNF expression (Figure 2(A,C)) compared to SCI mice. Meanwhile, our data revealed that treatment with WFA unregulated the expression of GDNF in spinal cord (Figure 2(B,D)).

### WFA inhibited the expression of Nogo-a and RhoA in spinal cord

**Figure 3.**
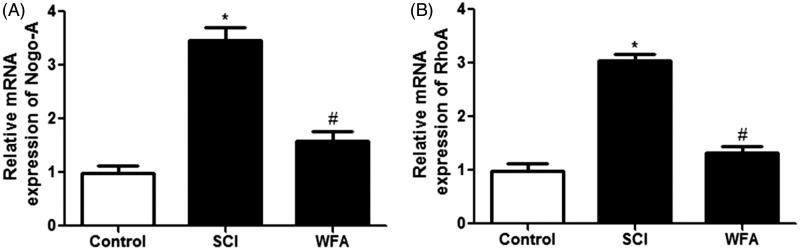
WFA inhibited the expression of Nogo-A and RhoA in spinal cord. The mRNA levels of (A) Nogo-A and (B) RhoA in spinal cord in SCI mice treated with saline or WFA (10 mg/kg) were determined with real-time PCR. *p < 0.05, compared with control mice; #*p* < 0.05, compared with SCI mice.

Next, the effects of WFA on neurite outgrowth inhibitory gens were evaluated using real-time PCR. Compared with the control, mice in SCI group showed an increased expression of Nogo-A and RhoA. However, we found that treatment with WFA obviously suppressed the mRNA expression of Nogo-A (Figure 3(A)) and RhoA (Figure 3(B)) spinal cord.

### WFA treatment reduced apoptotic cells in SCI mice

**Figure 4.**
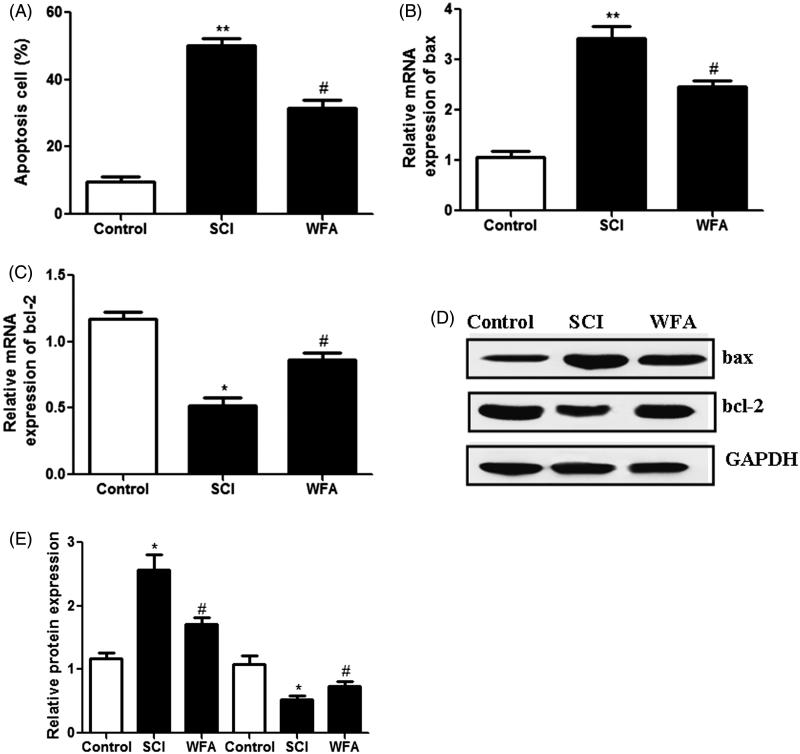
WFA treatment reduced apoptotic cells in SCI mice. (A) Detection of apoptotic cells in control mice and SCI mice treated by saline alone or WFA. Real-time PCR was used to measure the mRNA levels of (B) bax and (C) bcl-2. (D and E)The protein expression of apoptosis-related molecules was determined with western blot. *p < 0.05, **p < 0.01, compared with control mice; #*p* < 0.05, compared with SCI mice.

The effects of WFA on apoptotic cell death were determined in SCI mice using TUNEL assay. Compared with the control group, the average rate of apoptotic cells was significantly enhanced in SCI group. However, pretreatment with WFA obviously reduced the apoptotic cells (Figure 4(A)). In addition, we measured the expression levels of apoptosis-related proteins. Real-time PCR showed that administration of WFA led to the obvious down-regulation of pro-apoptotic protein bax (Figure 4(B)) and up-regulation of anti-apoptotic protein bcl-2 (Figure 4(C)). Western blot analysis also suggested that treatment with WFA partially reversed the SCI-induced up-regulation of bax and down-regulation of bcl-2 (Figure 4(D,E)). Collectively, these data indicated that WFA protected mice against apoptosis induced by SCI.

### WFA alleviated inflammatory responses in SCI mice

**Figure 5.**
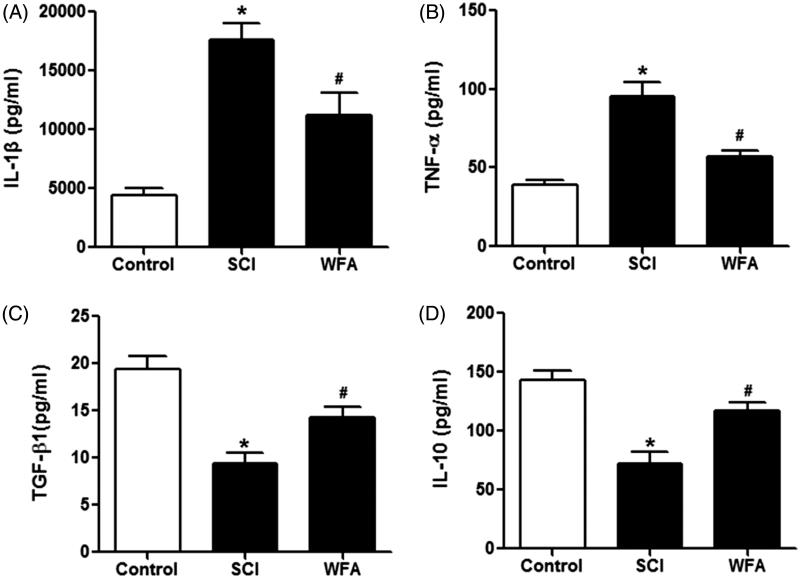
WFA alleviated inflammatory responses in SCI mice. ELISA was performed to determine the expression of (A) IL-1β, (B) TNF-α, (C) TGF-β1 and (D) IL-10 in SCI mice treated by saline alone or WFA. *p < 0.05, compared with control mice; #*p* < 0.05, compared with SCI mice.

In order to measure the anti-inflammatory role of WFA, we detected the expression of cytokines in mice. ELISA assay revealed that the expression levels of IL-1β and TNF-α were higher in SCI group than those in control group. However, the treatment of mice with WFA remarkably suppressed the production of IL-1β and TNF-α (Figure 5(A,B)). In addition, we examined the expression of anti-inflammation-related cytokines including TGF-β1 and IL-10. SCI mice exhibited decreased expression of TGF-β1 and IL-10, which were reversed in the presence of WFA pretreatment (Figure 5(C,D)). Taken together, these data suggested that WFA administration could attenuate the inflammatory response induced by SCI.

## Discussion

SCI is a major cause of disability in young people with high incidence and morbidity. Its consequences are devastating and lifelong, bringing about great suffering and heavy burden to the patients and their family. The current therapeutic efficacy for SCI is still not satisfied although a series of treatment strategies against SCI have been developed. Hence, it is urgent to find a novel and effective treatment for SCI. In this study, we demonstrated that WFA exhibited a neuroprotective role via regulation of apoptotic cell death and inflammatory responses in SCI mice.

It has been reported in several studies that *Withania somnifera* is an important medicinal herb with diverse therapeutic effects and wide clinical applications on cancer, anxiety, microbial infection and neurodegenerative disorders (Bhattacharya et al. 2000; Dar et al. 2016; Kataria et al. 2016). There has been different pharmacological evidence showing that WFA, a bioactive component of *Withania somnifera*, is a potential anticancer agent by regulation of reactive oxygen species (ROS) generation, p53 stabilization, p38 MAPK signalling activation (Yang et al. 2007; Mandal et al. 2008; Munagala et al. 2011). In addition, a previous study revealed the neuroprotective effect of WFA in central nervous system via down-regulation of stress response and pro-inflammatory mediators (Martorana et al. 2015). In our study, we found that treatment with WFA could improve neurobehavioural function and alleviate the histological alteration in spinal cord tissues in SCI mice. The production of BDNF and GDNF could alleviate inflammatory response and promote the functional recovery in acute SCI (Wu et al. 2008; Weishaupt et al. 2012). Meanwhile, some factors are able to inhibit the nerve regeneration such as Nogo-A and RhoA (Xing et al. 2011; Zhao et al. 2013). Our results demonstrated that WFA intervention led to increased expression of BDNF and GDNF and reduced Nogo-A and RhoA. Taken together, these data suggested the neuroprotective role of WFA in SCI mice.

The secondary damage followed by SCI, to a large extent, determines the outcome of SCI. It is involved in a series of cellular and molecular changes induced by the primary trauma, including the apoptotic cell death of neurons and glial cells (Xing et al. 2011; Oliveira et al. 2014). Our study indicated that treatment with WFA remarkably attenuated the apoptotic cells in SCI mice. Moreover, WFA injection resulted in a reduction of bax expression and an increase in bcl-2, suggesting the protective role of WFA against SCI-induced apoptosis.

Increasing evidence supports that SCI induces a robust immune response characterized by the production of chemokines and cytokines (Liu et al. 2013a). SCI-induced inflammation delays the functional recovery because of the scar tissue and cell apoptosis (Liu et al. 2013b). Accordingly, a variety of studies point out that inhibition of inflammation improves the locomotor function and promotes the spinal cord repair following SCI (Bracchi-Ricard et al. 2013; Guo et al. 2014). In the current study, we found that WFA intervention led to down-regulation of IL-1β and TNF-α and up-regulation of TGF-β1 and IL-10. Collectively, these data suggested that WFA treatment could attenuate the inflammatory responses induced by SCI.

In conclusion, the present study demonstrated that WFA-mediated inhibition of apoptosis and inflammation could protect against SCI. These findings suggest that WFA is a potential medication and deserves translational research for SCI therapy.
